# Risks and Protective Factors of Hispanic Families and Their Young Children during the COVID-19 Pandemic

**DOI:** 10.3390/children9060792

**Published:** 2022-05-27

**Authors:** Natasha Cabrera, Minxuan He, Yu Chen, Stephanie M. Reich

**Affiliations:** 1 Department of Human Development and Quantitative Methodology, University of Maryland, College Park, MD 20742, USA; ychen189@umd.edu; 2 Department of Psychology, Mount St. Mary’s University, Emmitsburg, MD 21727, USA; m.he@msmary.edu; 3 School of Education, University of California, Irvine, CA 92697, USA; smreich@uci.edu

**Keywords:** COVID-19, parental stress, parental engagement, socioemotional problems and skills, positivity, coparenting support

## Abstract

This study examines the risk-related factors during the pandemic and protective factors that might reduce its effects on family functioning in a sample of 161 low-income Hispanic parents in the United States, recruited from an ongoing longitudinal intervention study. They were surveyed about family functioning six months into the pandemic. We focused on the associations between social (e.g., exposure to the virus) and economic (e.g., job loss) pandemic-related risks on parental stress, parenting, and children’s socioemotional problems and skills, as well as the degree to which coparenting support, parents’ positivity, economic support, and access to services and information mitigated (protected) the negative effects of these stressors on family functioning. We found that increases in economic risk were associated with more child competence skills, whereas increases in social risk were associated with less parental engagement. Positivity and economic support moderated the effects of economic risk on parental stress and engagement. These findings show that to intervene effectively with low-income Hispanic families, we need to strengthen and support the resources for coping with adversity.

## 1. Introduction

The ongoing COVID-19 crisis continues to cause havoc in all aspects of our lives. The effect of the pandemic on family functioning and the measures in place to contain its spread have been particularly felt among families with limited economic and social resources, such as Hispanic families [1,2,3,4]. The pandemic has also dramatically changed young children’s lives; their daily routines and child care arrangements suddenly changed overnight [5,6]. Prior to the COVID-19 pandemic, 15.7% of Hispanic adults lived in poverty defined by the federal poverty threshold [7]. During the pandemic, almost 49% of Hispanic adults reported that they or someone in their household experienced a job loss or a cut in pay [8]. Data from the 2020 Current Population Survey show that poverty rates for Hispanic children rose by 4% during COVID-19, and that immigrant families were hit the hardest (36.3% to 42.2% from 2019 to 2020) compared to native-born families and families who are naturalized citizens [9]. Although there have been numerous reports and journal articles detailing the impact of the COVID-19 pandemic on families’ wellbeing, most of them have focused, and rightly so, on the economic consequences of the pandemic, and less on the short- and long-term effects on other aspects of family functioning, such as parenting, a critical driver of child development. Moreover, how the pandemic has impacted Hispanics, in particular, is still unclear [10]. The accumulation of risk induced by the pandemic—unemployment, disruption to child care, health issues, and even death—has added enormous new stress to parents who already experience economic stressors. The accumulation of stressors may further compromise parents’ ability to provide supportive and loving environments for their children [11,12,13,14]. 

Amidst adversity and chaos, low-income families also exhibit many strengths; that is, they draw on individual-, family-, or community-level resources to protect themselves from the negative effects of risk on their wellbeing [15]. For instance, being optimistic and having a good coparenting relationship with one’s partner can help parents cope with stress, feel less stressed, and engage with children in positive ways [2,16]. Economic support (e.g., the Supplemental Nutrition Assistance Program, SNAP) and having access to services and information about resources (e.g., information about parenting programs) are significantly related to parent and child wellbeing [17]. However, this literature is rather limited—there is less clarity about the protective factors that lessen the negative effect of hardships on families and help promote resiliency among children, especially among Hispanic families [18,19]. Thus, understanding the resources that families can draw on to protect themselves from harm can give us insight into the ability of that support system to protect them. More importantly, it can provide guidance to programs and policies to allocate resources in a way that actually meets the needs of families [15]. 

This paper focuses on a sample of economically diverse Hispanic families, a group that is underrepresented in resilience research, and examines the unique consequence of economic risk (i.e., job, income loss, and inability to make ends meet) and social risk (i.e., exposure to the virus and disruption in child care arrangements) experienced during the pandemic for parental stress, parental engagement, and children’s socioemotional problems and skills. We define social risk as factors that can disrupt social interactions with others and increase negative outcomes. Being exposed to the virus jeopardizes healthy social interactions with loved ones and others in the community. For instance, being exposed to the virus caused disruption in child care arrangements and relationships between parents and their children or other caregivers who must step in to provide care. Socioemotional skills (e.g., recognizing emotions in others and acting accordingly, understanding one’s thoughts and feelings) are essential for connecting with others and helping children manage their emotions, build healthy relationships, and feel empathy. 

Based on the empirical evidence, we also consider whether factors at the individual (e.g., parents’ positivity), family (e.g., supportive coparenting relationship), and community (e.g., access to services and information and economic supports) levels mitigate the negative effects of economic and social risks on family functioning among economically diverse Hispanic families [20,21]. We address the following research questions: (1) How are the economic and social risks experienced during the pandemic uniquely associated with mothers’ and fathers’ stress, parenting behaviors, and children’s socioemotional problems and skills? (2) How do perceived coparenting support, parents’ positivity, and economic support and access to services and information moderate the association between social and economic risks and parental stress, parenting behaviors, and children’s socioemotional problems and skills? 

### 1.1. Theoretical Background

The risk perspective hypothesizes that psychological or social factors increase the likelihood that an individual will experience poor outcomes [22]. High levels of economic and social risks are associated with a higher number of depressive symptoms and reduced parent–child engagement, which in turn are related to children’s behavioral problems [1,23,24]. The higher the number of risk factors children are exposed to at home, the higher the risk for behavioral problems [25]. Parents’ levels of risk are the strongest predictors of poor outcomes for children [3,26,27]. Although the negative effect of risk on families is a universal phenomenon, the levels of social and economic risks brought about by the pandemic have not been experienced equally, revealing pre-pandemic inequities and inequalities [28,29,30]. The pandemic has caused job loss, loss of income, and increased financial strain that have exponentially increased the levels of economic risk of families already living in very difficult circumstances [31,32]. Families with fewer economic and social resources have borne the brunt of it and the economic and health impacts on their wellbeing are beginning to be known [1,13,28,29,33,34]. Most of the pre-pandemic studies on risk only included maternal levels of risk and did not consider paternal levels of risk [27], and this is also true during the pandemic. Thus, in this paper, we include both mothers’ and fathers’ reports of social and economic risks.

We also draw from a relational developmental systems framework that families are systemic units of interlocked interactions and behaviors where people (e.g., mothers, fathers, and children) relate to one another separately and in dyads or subsystems (i.e., parent–child subsystem) [35]. For children, the development of resilience depends on the interactions with parents, who are key drivers of development. The central aspect of resilience is the identification of “protective processes,” which include characteristics of the individual, family, or community that lessen the detrimental effects of risk on family functioning [19,35,36,37]. In this paper, we focus on the early childhood period because it is most sensitive to environmental input, and both risks and protective factors are likely to have an impactful effect on brain development [38]. Given the centrality of the family for Hispanic families’ collective wellbeing, we focus on two potential protective factors: coparenting support and parent positivity. Because public investments play a big role in the lives of families and children, we also test whether community-level economics and access to services and information protect families from risk.

### 1.2. Family Functioning

Family functioning is a broad term that includes the social and structural characteristics of the family, as well as connections within the family. It also includes individual characteristics that enable members of the family to function as a cohesive unit [39]. An abundance of data show that parenting that is responsive to children’s needs and emotionally supportive is the main driver of children’s development [40,41]. Parenting is compromised when parents experience multiple risk factors that can make them less sensitive and warm toward their children [42]. Although the loss of jobs and interruption in child care arrangements during the pandemic have given parents the opportunity to spend more time with their children, the resulting income loss and change of routines for their children have also led to more economic hardship and more unstructured and chaotic time at home, which may increase stress and reduce the quality of parent–child interactions [42]. 

*Parental Stress.* Parents’ mental health is key to family functioning [43]. Individuals who perceive a lack of control over their lives and are always worrying are at risk for increased stress that impedes their ability to interact positively with their children [12]. COVID-19-related stressors appear to compound the stress already experienced by economically vulnerable parents in the U.S., further compromising mental health [44]. Research conducted during the COVID-19 pandemic shows that parents with young children are reporting increased levels of parenting stress [45,46]. 

*Child Social-Emotional Functioning.* One of the goals of families is to rear children who are socially and emotionally well-adjusted. Socioemotional skills (e.g., developing and supporting relationships with others, and expressing feelings and emotions) during early childhood are foundational to learning and social adjustment [47,48]. Parents who are affectionate, nurturing, and engage in activities that give children the opportunity to learn, share, listen, and resolve conflicts promote the development of socioemotional skills and prosocial behaviors [49]. Parental stress is particularly concerning because it can disrupt parents’ ability to be warm and nurturing with enduring negative consequences for children [48]. We focus on how pandemic-related social and economic risks are related to indicators of family functioning such as parental engagement in activities with children, parental stress, and children’s socioemotional skills.

### 1.3. Family Functioning in the Context of Risk

Risk, that is, the elevated probability of suffering harm or loss that could result in a negative outcome, has short- and long-term consequences on maternal and paternal stress, parenting practices, and children’s socioemotional skills [32,50,51]. Parents with a number of risk factors are at heightened risk for negative parenting and, consequently, increased emotional and cognitive dysregulation in children [52,53,54,55]. We focus on economic and social risks because they can have devastating and widespread impact on families and because many public investments are focused on reducing the negative impact of these risks on families.

*Economic risk* includes job loss, loss of income, and struggling to pay one’s bills that threaten the economic stability of the family. Although the mechanism is unclear, parents who experience high levels of economic stress have children who are more socially maladjusted than parents who report lower levels of economic stress [30]. Research to date has shown that pandemic-induced economic risks have a significant impact on parents’ ability to provide financially for their children [1,13,33,34]. A study of 183 ethnically diverse parents with children under 18 years of age found that COVID-19 stressors (e.g., stressors related to physical health, relationship with partner and children, or children’s academic learning) were positively associated with parental perceived stress, which was in turn associated with child abuse potential [1]. Another study using a large sample of low-income parents with preschool-aged children revealed that parents with both job and income losses experienced more depressive symptoms, stress, and more negative interactions with their children than parents with job loss but not income loss [3]. 

Although it may seem counterintuitive, some studies with Hispanic families find that when families experience financial difficulty, children exhibit more social behaviors, including helping behaviors, being more communicative and affectionate than problematic behaviors, a finding not typically observed in studies with non-Hispanic children [56,57,58,59]. Part of the explanation lies in the socialization practices of Hispanic parents who teach their young children to be caring and nurturing; exhibit concern for others; and satisfy the needs and expectations of others [48,60,61]. In this study, we test whether increases in economic risk are related to parental stress and elicit social behaviors from children.

*Social risk* is defined in this study as the elevated probability of a bad outcome as a consequence of disturbance in individuals’ social interactions with others, including disruption in child care, and exposure to the virus. A large source of stress for parents of young children during the COVID-19 pandemic was the loss of their pre-pandemic child care arrangements [8]. For parents who were still working during the pandemic, loss of child care jeopardized their ability to work, creating more stress. Although loss of child care increased parent–child time at home, it also increased risk conditions such as being in crowded homes, sharing small living space with others, and planning formal and informal activities for their children, all of which can be a huge burden on parent stress. In a study during the pandemic of 405 parents, almost half reported high levels of depression and parenting stress as well as more child anxiety and behavioral problems [62]. In this study, we test whether increases in social risk are associated with less parental engagement and more child socioemotional problems.

### 1.4. Protective Factors and Family Functioning

When experiencing adversity, resilient individuals cope, adapt, and overcome hardship by using psychological, social, and economic resources [35]. Protective characteristics of the child, family, and broader environment buffer individuals from the negative effects of risk on their wellbeing [36,37,63]. Surprisingly, there is more information about the links between risk and poor outcomes than there is about promotive and protective factors and wellbeing [21]. Thus, information about the characteristics that are protective and at what levels of risk is quite limited [64,65]. In this study, we test whether or not protective factors—parents’ positivity, coparenting support, access to services and information, and community-level economic support—moderate the negative effects of social and economic risks on family functioning [20,21]. 

*Positivity*. Research conducted before the pandemic demonstrates that people who are highly optimistic have higher levels of psychological wellbeing, affirmative social relationships, and higher capacity to handle stressful situations than people who are less optimistic [66,67,68,69,70]. Mothers who report being highly positive also report lower levels of internalizing symptoms and higher levels of child adjustment [71,72]. Positivity has been related to effective parenting for mothers and fathers and to children’s socioemotional adjustment [73]. There is also evidence that positivity mitigates the negative impact of economic stress on parents’ mental health [71,72]. A study of a large sample of healthcare workers in Germany found that during the pandemic optimism was significantly related to lower levels of depressive and anxiety symptoms [21]. Based on this review of the literature, we test whether or not parents with higher levels of positivity would be more likely to engage in positive parenting in the face of heighted risk. 

*Coparenting* refers to couples’ capacity “to work together as a team” to fulfill their parenting responsibilities and has been found to promote family positive functioning [74,75]. Coparenting that is highly supportive is strongly associated with responsive parenting and children’s social outcomes [27,76,77,78,79,80]. Researchers in New Zealand found that the association between depression and negative quality of parenting during the pandemic was stronger only for couples who reported low levels of coparenting support [81]. Consequently, we examine whether the negative association between social and economic risks and family functioning is reduced when parents report higher levels of coparenting support.

*Economic support*. Federal programs, such as the Supplemental Nutrition Assistance Program (SNAP), are strongly related to better academic achievement [17,82,83]. Frongillo and colleagues (2006) showed that children who participated in food stamp programs from kindergarten to third grade had a 3-point increase in their reading and math scores compared to children who stopped participating during those four years [82]. In another study, SNAP participation significantly mitigated the negative association between economic hardship (e.g., difficulty getting by on family’s income) and children’s grade retention [17]. Improvement in families’ income has also been associated with better child outcomes [84]. In this study, we test whether economic support, in the form of unemployment insurance, SNAP/WIC, and access to food banks protected families from the negative effects of economic and social risks.

*Access to services and information* that validate and provide information have also been found to be protective against risk. A study with 205 women found that access to health information (e.g., sources of information, satisfaction with the quality of information, usefulness of information) was indirectly associated with women’s emotional and physical wellbeing through levels of depression and anxiety [85]. Other studies have also suggested that participation in parenting education programs and access to online parenting information may benefit parents’ wellbeing and children’s developmental outcomes [86,87]. Given these findings, we test hypothesis that having access to helpful information and needed services during the COVID-19 pandemic would mitigate the negative effects of risk on their family functioning.

### 1.5. Current Study

There is clear evidence that the pandemic has exacerbated the social and economic risks that Hispanic families experienced before the pandemic with potentially dire consequences for family functioning. We expand these findings by asking: (1) are pandemic-related economic and social risks associated with Hispanic mothers’ and fathers’ stress, engagement with their children, and children’s socioemotional problems and skills? (main effects)? (2) is the association between pandemic-related risks and parental stress, parental engagement, and children’s socioemotional problems and skills moderated by coparenting support, parental positivity, economic support, and access to services and information (moderation hypothesis)? Framed within a risk and resilience perspective, we hypothesize that higher levels of economic and social risks would be associated with increased parental stress, less engagement in activities with children, more child behavior problems, and fewer social competence skills (main effects). We also hypothesize that high levels of coparenting support and positivity would weaken the association between social and economic risks and family functioning, and that high levels of economic support and having access to services and information would also weaken this association (moderation effects).

## 2. Materials and Methods

### 2.1. Procedures

Participants were drawn from Baby Books 2 project (BB2 project), an NICHD-funded longitudinal intervention study that aims to provide child development information to first-time low-income parents [88]. Participating families were recruited from centers that administer the Specific Supplement Nutrition Program for Women, Infants, and Children (WIC), health care clinics, emergency department waiting rooms, parks, and community centers in both the Washington, D.C. metropolitan area and in Orange County, California. Eligible families for the BB2 project were (1) first-time parents of a baby aged 9 months, (2) cohabiting, (3) over the age of 18, (4) making less than $75,000 per year as a household, and (5) literate at a first-grade reading level in either English or Spanish. All infants were full-term (over 37 weeks of gestation), and all procedures and materials were approved by Institutional Review Boards at both universities.

BB2 participants received text messages during May and August 2020 asking them if they wanted to take part in a study about the COVID-19 pandemic. After giving consent to participate, parents received a personal link to access the online survey, administered by Qualtrics. Parents were given a maximum of 21 days to complete the survey in their preferred language, either in English or Spanish. All parents except for one completed the survey on their phone. A total of 292 parents were contacted and 247 consented and completed this survey (84.6% of response rate). Data collection took place from July 2020 to September 2020. After the completion of the survey, participants received a USD 20 e-gift-card as compensation and were entered into a draw for one of four USD 50 e-gift-cards. Since individual links were sent to each participant, participants did not enter any identifiable information during this survey. The survey took an average of 31 min to fully complete. Because this paper focuses on Hispanic families, we included families who self-identified as Hispanic, resulting in an analytic sample of 161 parents from 95 families: 132 parents were a couple, 26 mothers and 3 fathers were single respondents whose partners did not participate in the survey.

### 2.2. Participants

Participating parents who self-identified as Hispanic had children ranging from 22 to 55 months in age (Mean age = 2.9 years, SD = 0.5) at the time of this study. The sample consisted of 40 boys (42%) and 55 girls (58%) and of more mothers (57%; Mean age = 29.8 years old, SD = 6.0) than fathers (43%; Mean age = 32.1 years old, SD = 6.4). Thirty-nine percent of the participants (n = 63 parents) resided on the east coast in the Washington, D.C., Virginia and Maryland, and 61% resided on the west coast in the Orange County, California. There were no significant differences in household income or educational levels between the analytic sample and the full BB2 sample. The pre-pandemic average yearly household income was USD $39,934 (SD = 20,721). Sample demographics and descriptive data of study variables are presented by parent gender in Table 1. 

### 2.3. Measures 

This study examined five family outcomes, including mothers’ and fathers’ stress levels, parental engagement, and children’s socioemotional problems and socially competent behaviors. Our predictor variables included pandemic-induced economic and social risks. The economic risk index consists of parent reports of changes in employment (loss of job or hours) and financial ability to make ends meet (e.g., rent, utilities, groceries) since the COVID-19 pandemic began. The social risk index consisted of parent reports of exposure to the SARS-CoV-2 virus, and difficulty in accessing child care. We also included four moderators: parent positivity, coparenting support, economic support, and parents’ access to services and information during the pandemic. All variables were averaged to create family-level variables, except for parental stress and parental engagement. Reports from single-respondent families were used as parent scores. All variables had 5 levels or more and were used as continuous variables for later analyses. Detailed descriptions of these variables are listed below. 

#### 2.3.1. Outcome Variables

*Child socioemotional problem behaviors* were assessed using modified questions from the problem behaviors subscale of the Brief Infant and Toddler Socioemotional Assessment (BITSEA) [89]. Mothers and fathers were asked to report perceived changes in children’s problem behaviors since the COVID-19 pandemic began. Answer choices of the original questions used a 5-point Likert scale (1 = “a lot less”, 2 = “a little less”, 3 = “the same”, 4 = “a little more”, 5 = “a lot more”, and “does not apply”). Questions attempted to capture changes in five types of behavioral problems: (1) “been having tantrums and angry outbursts”; (2) “been struggling to manage their emotions”; (3) “been engaging in aggressive behavior such as hitting, biting, scratching and throwing objects…”; (4) “been crying”; and (5) “been needing to be held”. Items marked as “does not apply” received a score of 0. Ratings of the five items were averaged and ranged from 0 to 5. Higher scores indicate more behavioral problems since the pandemic began. Internal consistency was good (Cronbach’s alpha = 0.86).

*Child socially competent behaviors* were assessed using modified questions from the competence subscale from the BITSEA. Social competence scores included three questions rated on the same 5-point scale (1 = “a lot less”, 2 = “a little less”, 3 = “the same”, 4 = “a little more”, 5 = “a lot more”, and “does not apply”). Questions attempted to capture changes in three social competence skills: (1) “been talking/communicating with you”; (2) “been wanting to help”; (3) “been affectionate (e.g., gives hugs, uses caring words, etc.).” Likewise, “does not apply” received a score of 0. Ratings of the three items were averaged and ranged from 0 to 5. Higher scores indicate more socially competent behaviors since the pandemic began. Internal consistency for this measure was adequate (Cronbach’s alpha = 0.65). 

*Parental stress* during the pandemic was assessed using the Perceived Stress Scale (PSS) [90]. Parents were asked to report how much they experienced stressful situations in the past two weeks on a 4-pt Likert scale (0 = “Never”, 1 = “Sometimes”, 2 = “Fairly often,” and 3 = “Very often”). The scale included four items: (1) “you were unable to control the important things in your life”; (2) “things were going your way”; (3) “confident about your ability to handle your personal problems?” and (4) “difficulties were piling up so high…”. Two items were reverse coded and ratings of these items ranged from 0 to 12. Higher scores indicate feeling more stressed. Internal consistency for this measure was acceptable (Cronbach’s alpha = 0.71). Mother stress and father stress scores were treated separately because they were important indicators of individual functioning and paired sample tests showed that mother stress and father stress was not correlated and significantly differed from each other (r = −0.06, *p* < 0.05).

*Parental engagement* was assessed with five items on a 6-point scale (1 = “not at all”, 2 = “rarely”, 3 = “a few times a month”, 4 = “a few times a week”, 5 = “about once a day”, 6 = “more than once a day”). Items attempted to capture specific activities parents engaged in with their child since the COVID-19 pandemic began: (1) “Playing together”; (2) “putting the child to bed”; (3) “going for a walk together”; (4) “singing songs and telling stories”; and (5) “reading a book together”. Internal consistency was acceptable (Cronbach’s alpha = 0.72). Unlike past studies that used typically mothers, we used the sum score of mothers’ and fathers’ reports of parental engagement to underscore the total amount of “exposure” to parenting. Mothers’ and fathers’ self-reported ratings of engagement were correlated (Pearson r = 0.26). 

#### 2.3.2. Predictor Variables

We surveyed participants about possible changes in four stressful experiences in their economic and social life since the COVID-19 crisis began (adapted from Brailovskaia & Margaf, 2020) [91]. Table 2 shows both the number and percent of participants who reported various levels of adversities. An economic risk index was computed by summing response scores about income loss and inability to making ends meet. Similarly, a social risk index was computed by summing response scores about exposure to SARS-CoV-2 virus and difficulty in accessing child care. 

*Economic risk* was assessed by two items: (1) job or income loss; and (2) financial difficulty in making ends meet. Job or income loss asked about changes in participants’ employment status since the pandemic began. Participants who reported “no change” or “got new job/gained hours” were coded as 0 indicating no difficulty in employment while those who reported “lost job/lost hours” were coded as 1 indicating some difficulty in employment. We also asked about changes in the ability to make ends meet with two questions about their ability “to pay bills (e.g., rent, utilities)” and “to buy basic needs (e.g., food, diapers).” For each item, when participants reported some level of difficulty (e.g., “Yes, it is slightly more difficult”, or “Yes, it is much more difficult”), they were scored as 1. When participants reported no difficulty (e.g., “No change”, or “Yes, it is easier than before”), they were scored as 0. Each participant’s scores of job or income loss and financial struggles were added up to create an economic risk index at the individual level ranging from 0 to 2 with 0 indicating no economic stress, 1 for one economic risk, and 2 for two economic risks. We then created family-level risk scores ranging from 0 to 2. The family-level economic risk scores can be interpreted as follows, since the pandemic began: score = 0, neither parent reported changes in job/income or financial ability; score = 0.5, one parent reported loss in either job/income or financial ability; score = 1, one parent reported loss in job/income and financial ability or both parents reported one negative change; score = 1.5, one parent reported a negative change and the other parent reported two negative changes; score = 2, both parents reported negative changes in job/income and financial ability. 

*Social risk* was also assessed with two items: (1) exposure to SARS-CoV-2 virus, and (2) disruption in child care. Exposure to SARS-CoV-2 virus asked the participants if they “have tested positive myself” or their close contact (“Someone with whom I live or work tested positive”) had been diagnosed as COVID-19 positive. The answers from both questions were merged in a single variable, named “Exposure to virus”. Participants were considered not exposed (0 = no exposure) if they reported “My physical health has not been affected” and also “The health of those close to me has not been affected.” Otherwise, they were considered as being exposed to the SARS-CoV-2 virus (1 = exposure, e.g., not positive themselves but people close to them were infected). Disruption to child care asked parents about changes in their access to child care since the pandemic. If “no change” or “easier than before” were selected, parents received a score of 0. If “slightly more difficult” or “much more difficult” was selected, they scored 1 for this measure. Each participant’s scores of virus exposure, and child care disruption were added up to create a social risk index at the individual level ranging from 0 to 2 with 0 indicating no social stress, 1 for one social risk, and 2 for both social risks. We then created family-level social risks scores ranging from 0 to 2 and can be interpreted as follows, since the pandemic began: score = 0, neither parent reported exposure to virus, nor disruption to child care; score = 1, one parent reported exposure to virus and disruption to child care or both parents reported one adversity; score = 2, both parents reported exposure to virus and child care disruption. 

Therefore, both family-level economic and social risk index ranged from 0 to 2 with 5 possible levels. They were entered as continuous variables in later analyses. 

#### 2.3.3. Moderator Variables

*Parent positivity* assessed parents’ self-esteem, life satisfaction, and positivity with 6 items from the Positivity Scale (P Scale) [92]. Sample items include “I have great faith in the future” and “I feel that others are generally here for me when I need them”. Participants rated their agreement on a 5-point Likert scale (1 = “strongly disagree” to 5 = “strongly agree”). One item (“At times, the future seems unclear to me”) was reverse coded. The total score ranges from 6 to 30. Higher scores were associated with more optimism or confidence in the future. Internal consistency for this measure was good (Cronbach’s alpha = 0.79). 

*Coparenting support* assessed parents’ perception of support by the other parent in parenting activities with seven items from the Coparenting Support subscale of the brief Coparenting Relationship Scale (CRS) [93]. Sample items include “My partner and I have the same goals for our child” and “my partner appreciates how hard I work at being a good parent”. Participants rated their agreement on a 7-point scale (0 = “not true of us” to 6 = “very true of us”). Summary scores ranged from 0 to 42. Higher scores indicate more perceived coparenting support. Internal consistency for this measure was good (Cronbach’s alpha = 0.89). 

*Economic resources and access to services and information* were assessed by asking participants to report the type and number of support they received since the pandemic began from a checklist: (1) WIC/SNAP; (2) unemployment insurance; (3) food banks or school food pick-up; (4) healthcare or mental health services; (5) online resources for education; (6) online resources for exercise; (7) online websites for parenting; (8) cash advances on credit cards; (9) utility waivers or discount; (10) emergency child care; (11) domestic violence shelters; (12) emergency loan or borrowed money; (13) other; (14) none of the above. We focus on government-sponsored assistance in this study. Of these, three are government-sponsored economic assistance: WIC/SNAP, unemployment insurance, food banks or school food pick-up. The rest of the items (4–13) were about resources for parents to access services and information. We then created family-level support scores by aggregating the number of resources reported by both parents. Therefore, the family-level economic support scores ranged from 0 to 3 and accessing services and information scores ranged from 0 to 4. 

### 2.4. Analytic Plan

Our analysis is based on an analytic sample of n = 95 Hispanic families (92 mothers and 69 fathers). Less than 2% of data were missing at the family level, including one missing score for parent positivity and one for coparenting support. We used path analysis with maximum likelihood (ML) method to calculate estimators using RStudio 1.2.5 (RStudio Team (2020). RStudio: Integrated Development for R. RStudio, PBC, Boston, MA URL http://www.rstudio.com (accessed on 1 February 2022). (PBC, Boston, MA, USA). The model included 2 family-level predictors (economic and social risks), 5 outcomes (maternal stress, paternal stress, parental engagement, child socioemotional problems, and child social competence), and 1 control variable (highest education level in the family). Predictors and outcomes were allowed to covary in the model. To test for moderation effects, we added 6 interaction terms (e.g., economic risk × parent positivity, economic risk × coparenting support, social risk × parent positivity, social risk × coparenting support, economic risk × economic support, social risk × access to services and information) in the model. Both main effect and moderation effect models were saturated. The predictor and moderator variables were first mean-centered and then used to calculate the interactions to reduce multicollinearity among the study variables. We report standardized estimates of all estimators. Finally, we used simple slopes analysis to visualize the moderation interactions using Process v4.0 in SPSS 27 [94]. 

## 3. Results

### 3.1. Descriptive and Correlation Analyses

Table 2 illustrates the incidence of each set of stressful experiences in individual parents. Since the pandemic began, the majority of participants (70%) experienced at least one economic risk and approximately 54% reported experiencing some social risk. Our study sample reported high levels of positivity (Mean = 22.5, SD = 4.2); high level of supportive coparenting relationship (Mean = 34.6, SD = 7.6); low levels of stress (Mean = 4.1, SD = 2.4 and Mean = 3.4, SD = 2.3, respectively); and, fathers reported a small increase in engagement with their child (Mean = 38.6, SD = 6.5). Compared to pre-pandemic time, parents observed little change in their children’s socioemotional problem behaviors (Mean = 2.5, SD = 1.0); and reported an increase in their children’s social competence (Mean = 4.1, SD = 0.7). Table 3 shows zero-order correlations of predictors, moderators, and outcomes.

**Figure 1:**
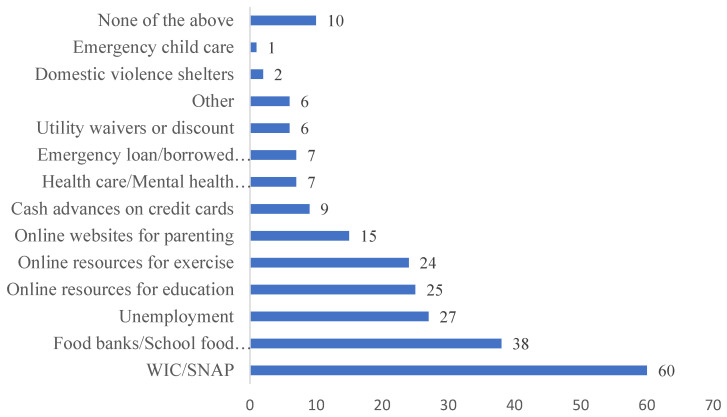
Economic, services, and resources used early on in the COVID-19 pandemic. Note. Number of families (n = 95) who reported use of each type of resources since the COVID-19 crisis began.

Figure 1 shows the number of services and economic-related information and support parents accessed during the pandemic. Among the 95 families in our sample, since the pandemic began 89% have accessed at least one source listed on the list. The most often used support parents accessed was WIC and SNAP (63% of the families used), followed by food supplies (40%) and unemployment compensation (28%). In addition, various online services and resources were used such as information on education (26%), fitness (25%) and parenting (16%). 

### 3.2. Path Analysis: Main Effects

**Figure 2:**
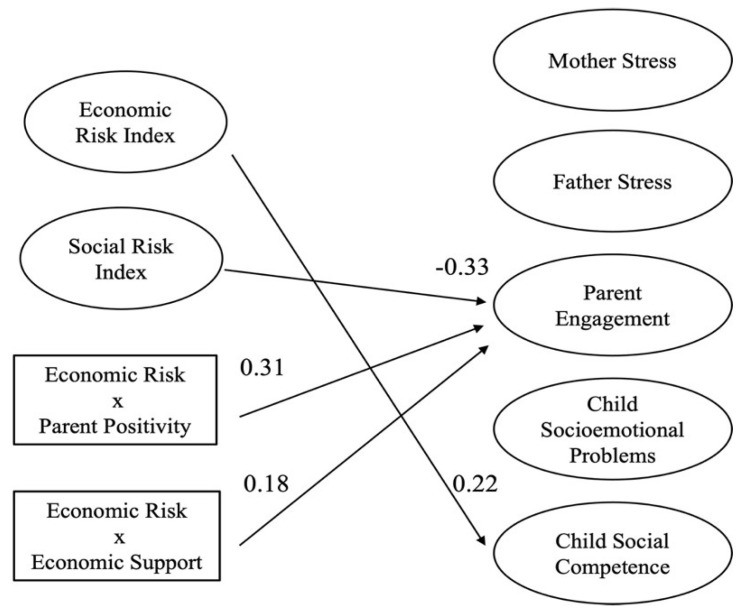
Associations between economic and social risks and family functioning. Note. All predictors are mean-centered. For parsimony, errors and non-significant coefficients are omitted from the figure. All standardized coefficients and covariances are significant at *p* < 0.05.

We conducted one path model to examine the associations between the economic and social risks experienced by Hispanic parents and the five family functioning outcomes (i.e., maternal and paternal stress, parental engagement, child socioemotional problems, and child social competence) and four protective factors (i.e., parent positivity, coparenting support, economic support and access to services and information; Figure 2).

We found that since the pandemic began and controlling for parent education, parents’ increase in economic risk was associated with parent reporting that their child was more socially competent (i.e., increased communication, was affectionate, and wanted to help). That is, a one standard deviation increase in economic risk was associated with a 0.22 standard deviation increase in child social competence scores (β = 0.22, 95% CI = [*0.03*, *0.42*], *p* < 0.05), keeping everything else constant. 

Reports of increase in social risk was associated with less parental engagement (β = −0.33, 95% CI = [−*0.49*, −*0.16*], *p* < 0.001). That is, a one standard deviation increase in social risk was associated with 0.33 standard deviation decrease in parental engagement scores, keeping everything else constant (see Figure 2). 

### 3.3. Moderation Effects 

To test for moderation effects, 6 interaction terms (economic risk x parent positivity, economic risk x coparenting support, social risk x parent positivity, social risk x coparenting support, economic risk x economic support, social risk x access to services and information) were added to the main effects model. We found two significant interactions. To further test the moderation effect, we used simple slopes analysis [94].

**Figure 3:**
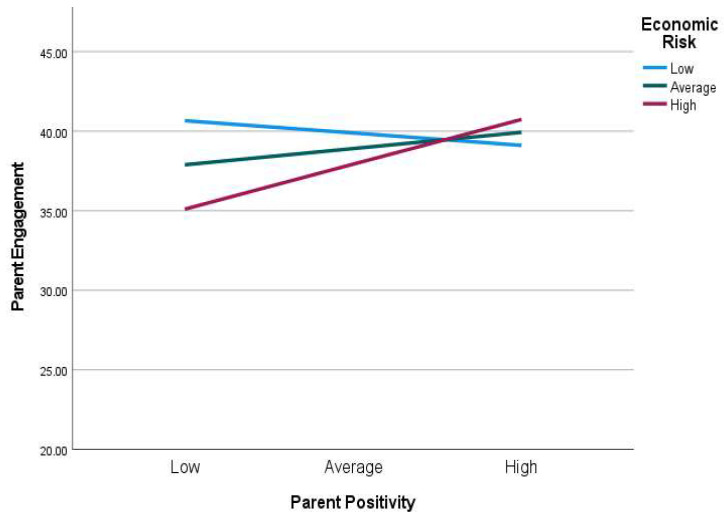
Parent positivity moderating the effect of economic risk on parental engagement scores. High = 1 standard deviation above the sample mean, average = sample mean, and low = 1 standard deviation below the sample mean.

Parent positivity during the pandemic moderated the association between economic risk and parental engagement, β = 0.31, 95% CI = [*0.12*, *0.50*], *p* < 0.01. When parents reported low level of positivity, parental engagement was lower than when parents reported average and higher levels of positivity. Therefore, parent positivity protected families from the negative impact of economic risk on parental engagement during the pandemic. The protective effect was strongest at high level of parent positivity (Figure 3).

**Figure 4:**
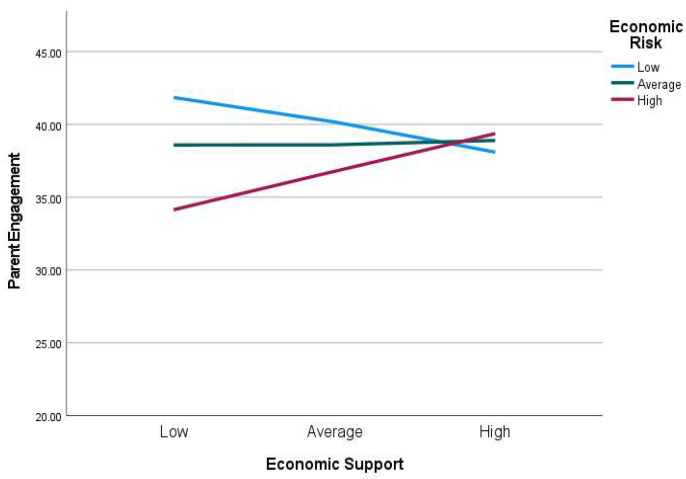
Economic support moderating the effect of economic risk on parental engagement scores. High = 1 standard deviation above the sample mean, average = sample mean, and low = 1 standard deviation below the sample mean.

Economic support received during the pandemic moderated the association between economic risk and parental engagement, β = 0.18, 95% CI = [*0.00*, *0.36*], *p* < 0.05. When families received high level of economic support, parent reported more engagement than those who received low level of economic support. Therefore, economic support protected families from the negative impact of economic risk on parental engagement, especially under high levels of economic support (Figure 4).

## 4. Discussion

Two years into the pandemic, its costs continue to be felt by some people more than others [13,95]. Economically vulnerable families have suffered more substantially and consequently have experienced most dire and long-lasting effects [7,8]. Using survey data from the BB2 study, we explored how economic and social risks during the pandemic related to Hispanic family functioning, including parents’ stress, parental engagement, and children’s socioemotional skills. We also considered how family characteristics—optimism and coparenting support—and community-level resources such as economic support and access to services and information not only promoted family functioning but also protected families against the negative impacts of the pandemic and helped them be resilient during this crisis. In considering both risks and protective factors, this study can guide policy and programs to acknowledge and validate the resources and assets that families have and to do so thoughtfully and carefully to strengthen their resilience and maximize the impact of the support they receive. First and consistent with other findings, our data show that six months into the pandemic approximately 30% of low-income Hispanic parents reported both job/income loss and inability to make ends meet [1,83]. Almost 30% of our participants have had some exposure to the virus and almost 40% had no access to child care (see Table 2). Given that the data were collected in the summer of 2020, the rates of COVID-19 exposure are likely to be higher now. In fact, recently released reports show that Hispanics experienced a disproportionate number of cases and fatalities in the U.S. [96]. 

We report several significant findings. First, we find that half a year into the pandemic, parents reported increases in social (e.g., child care loss, and exposure to the virus) and economic (i.e., job loss and inability to make ends meet) risks. Yet, parents also reported relatively low levels of stress and high levels of engagement with their children. In addition, parents reported that since the pandemic started their children have behaved more socially (e.g., wanted to help, were more communicative and affectionate). Contrary to our hypothesis and to past findings, economic risk was not significantly associated with less family functioning [3]. Unexpectedly, high levels of economic risk were associated with higher levels of reported social competence in children. This counterintuitive finding is consistent with studies that have shown that in Hispanic families, children are socialized to respond with concern and love when they see someone in distress [56,57]. In a recent study of parents of children of 8 years and younger, COVID-19 pandemic-related financial and mental health stresses were associated with increases in children’s prosocial behaviors [59]. Hispanic children who are socialized to be caring and sensitive towards others seem to act more socially during difficult times. We did not find support for our hypothesis that increased economic risk would result in increased stress. One possible explanation is that in our study families reported relatively low to average levels of economic risk (i.e., the average score was 1 that is one or both parents reported changes in employment status and financial ability).

Second, we found partial support for the hypothesis that increase in social risk would be associated with negative family functioning. Six months into the pandemic-induced social restrictions, being exposed to the virus or having no access to child care for their young children did not significantly increase parents’ stress or their perception that their children misbehaved more, but it reduced the amount of time they spent with their children in fun and enjoyable activities such as playing or reading. The stress caused by the lack of child care and exposure to the virus likely depleted parents’ energy and motivation to engage with their children. This is concerning because parental engagement, especially during difficult times, is critical for children’s wellbeing [97]. 

Third, we found no evidence that our hypothesized promotive factors shielded parents from the negative effects of social risk on family functioning, but we found evidence that two promotive factors—positivity and economic support-- protected them from the negative effects of economic risk on parenting. That is, parents with high levels of economic risk reported less parental engagement with their children only when they also reported low levels of positivity and low levels of economic support. At higher levels of positivity and economic support, parents reported more engagement with their children when they also reported increases in economic risk. These findings are consistent with studies showing the protective effect of economic support (e.g., WIC, SNAP, food banks) on children and families [17,82]. It seems that for Hispanic families receiving economic support and being positive are important mechanisms that ensure children are protected from the economic hardship their parents experience.

Although not central to our hypotheses, there are a couple of findings worth discussing because they help contextualize the main findings. Parents with high level of positivity reported significantly low level of parental stress (although the direction is unclear) and parents who perceived their coparenting support to be strong also reported more engagement with their children. These aspects of families—coparenting and parents’ positive outlook on life—cannot be underscored enough as these not only promote wellbeing but also in the case of positivity protect families from harm, indicating a possible reason why families our study were able to somewhat protect their children during these stressful times.

### Limitations

There are several limitations to this study. First, a cross-sectional design is not ideal to show the directions of associations. It is possible that the associations were reversed of what we found, that is, Hispanic families who showed lower levels of family functioning would be more prone to economic and social risks related to the pandemic. Longitudinal studies are needed to better understand the underlying mechanisms through which pandemic-related risks relate to family functioning and child outcomes. Second, several scales were adapted from the original and did not include all the items. Those scales were shortened to reduce the burden on parents in our sample, who were already going through a difficult time during the pandemic and were very reluctant to participate in any study. Therefore, it is not possible to compare these data with the norms established by the original scales and the content validity of the scales may be compromised because the omitted items may capture important information on the concept of interest. One way to validate these abbreviated scales is using item response theory to assess their reliability. Third, due to restrictions on in-person visits and constraints on time, logistics, and funding, we were not able to directly measure children’s behaviors through observations and standardized assessments. Although we considered both parents’ reports to calculate children’s socioemotional problems and skills, thus reducing the measurement error to a certain degree, parents’ report of children’s behaviors can be still biased. Fourth, our study could not include all relevant questions that might have helped us to get a better picture of family functioning during the pandemic. So, it is difficult to rule out factors others than the ones included in this study that may influence child behaviors. Longitudinal designs are better able to address this uncertainty. 

## 5. Conclusions 

Since the pandemic began, Hispanic families have experienced their share and more of social and economic shocks: loss of job and income and the uncertainty that working may increase the chances of contracting the virus and infecting themselves and their families. Amidst all this, these families also lost their child care arrangements making it impossible to keep a job and provide for their families. Yet, in the middle of this crisis, we also found that Hispanic families did their best to protect their children from the chaos around them and keep them engaged and happy at home. Six months into the pandemic despite families reporting reduced work hours and financial adversities, they also reported relatively low levels of stress, high levels of optimism and engagement with their children, and reported that their children were not misbehaving but actually were kinder and more helpful. This is an important finding that during economic crisis, Hispanic young children display more social behaviors that ultimately must help parents deal with the crisis. This finding speaks to the strength and resilience of the entire family. What is helping families? What is protecting them from the harm that the pandemic inflicted on their lives? We found evidence that both economic support and levels of optimism were important protective mechanisms. Over 60% of the families reported using at least one economic resource such as WIC/SNAP. High levels of economic support and being optimistic protected families from the negative effect that economic risk had on their ability to spend time with their children. Families who experienced high levels of economic hardship were able to stay engaged and involved with their children when they received high levels of economic support such as WIC/SNAP and felt optimistic about the future. Although we need longitudinal designs, the findings of this study point to two important points of intervention for families that build on what they are doing already: economic support and mental health services to support and strengthen their strong positive outlook in life. Future studies should also explore other forms of economic and social support at individual- and family- levels. This study identifies potential structural and cultural strengths that help Hispanic families cope with and be more resilient during this unique time in history.

## Figures and Tables

**Table 1:** Description of mothers’ and fathers’ characteristics.

	Total			Mothers			Fathers		
Variable	n	%	M (SD)	n	%	M (SD)	n	%	M (SD)
Age	-	-	30.8 (6.3)	-	-	29.8 (6.0)	-	-	32.1 (6.4)
* Parents’ Education									
Less than HS	23	14	-	4	4	-	19	28	-
Completed HS	37	23	-	21	23	-	16	23	-
Some College	53	33	-	32	35	-	21	30	-
4-year degree or higher	48	30	-	35	38	-	13	19	-
Household Income	-	-	39,934 (20,721)	-	-	-	-	-	-
$0–$25.000	46	28	-	29	33	-	17	25	-
$25.000–$50.000	66	41	-	39	43	-	27	39	-
$50.000–$75.000	38	24	-	20	21	-	18	26	-
>$75.000	11	7	-	4	2	-	7	10	-
Total	161	97	-	92	98	-	69	96	-
	M (SD)		Range	M (SD)		Range	M (SD)		Range
Parental Stress	4.0 (2.5)		0–11	4.1 (2.4)		0–11	3.4 (2.3)		0–11
Parental Engagement	38.6 (6.5)		5–50	20.4 (3.1)		8–25	17.8 (4.2)		1–25
Child Socioemotional Problems	2.4 (1.2)		0–5	2.5 (1.2)		0–5	2.2 (1.2)		0–4.8
Child Social Competence	4.1 (0.9)		0–5	4.1 (0.8)		2–5	4.0 (0.9)		0–5
Economic Risk	1.0 (0.7)		0–2	1.0 (0.7)		0–2	0.9 (0.8)		0–2
Social Risk	0.6 (0.6)		0–2	0.7 (0.7)		0–2	0.6 (0.6)		0–2
Positivity	22.5 (4.8)		9–30	22.1 (5.1)		9–30	23.1 (4.3)		10–30
Coparenting Support	35.0 (8.0)		2–42	34.3 (8.2)		2–42	36.0 (7.6)		9–42
Economic Support	1.1 (0.7)		0–3	1.1 (0.8)		0–3	0.9 (0.8)		0–3
Services and Information	0.5 (0.8)		0–3	0.6 (1.0)		0–3	0.4 (0.9)		0–3

Note: Due to missing data on some study variables, not all responses to individual items sum to 161 individual parents. * Family level variables on this table are summed across both parents for family income, and highest degree in the family for parent education. HS = High school.

**Table 2:** Number and percent of individual parents encountering various economic and social risks.

Economic Risk Index	N (Total = 157)	%	Social Risk Index	N (Total = 158)	%
No economic risk	47	30.0%	No social risk	73	46.2%
Job loss without inability to make ends meet	20	12.7%	Exposure to virus without daycare disruption	27	17.1%
Inability to make ends without job loss	42	26.7%	Daycare disruption without exposure to virus	42	26.6%
Both job loss and inability to make ends meet	48	30.6%	Both exposure to virus and daycare disruption	16	10.1%

Note: Due to missing data on some variables, not all responses to individual items sum to 161 individual parents.

**Table 3:** Zero-order correlations for predictors, moderators, and outcomes.

	Study Variables	1	2	3	4	5	6	7	8	9	10	11
1	Economic risk	--										
2	Social risk	0.24 *	--									
3	Parent positivity	−0.18	−0.06	--								
4	Coparenting support	−0.13	−0.20 *	0.41 ***	--							
5	Economic support	−0.07	0.20	−0.02	−0.03	--						
6	Services and information	0.07	0.13	0.03	−0.09	0.10	--					
7	Mothers’ stress	0.23 *	0.14	−0.36 ***	−0.31 **	0.10	0.12	--				
8	Fathers’ stress	0.18	0.16	−0.37 **	−0.30 *	0.11	−0.08	−0.06	--			
9	Parental engagement	−0.18 ^+^	−0.38 ***	0.23 *	0.37 ***	−0.07	−0.01	−0.01	−0.24 *	--		
10	Child socioemotional problems	−0.05	0.12	−0.05	−0.18 ^+^	0.16	0.24 *	0.35 ***	−0.21	0.08	--	
11	Child social competence	0.08	−0.04	0.29 **	0.20 ^+^	−0.07	−0.08	−0.11	−0.18	0.08	0.07	--
	Mean	1.0	0.6	22.5	10.0	1.0	0.5	4.1	3.4	38.6	2.5	4.1
	SD	0.7	0.6	4.2	2.9	0.7	0.8	2.4	2.3	6.5	1.0	0.7
	Min	0	0	10	4	0	0	0	0	10	0.5	2
	Max	2	2	30	16.5	3	3	11	11	50	4.8	5

Note. Due to missing data on some variables, not all responses to individual items sum to 95 families. All variables were aggregated at family level except for parental stress 0.5 < ^+^ < 1.0, * *p* < 0.05, ** *p* < 0.01, *** *p* < 0.001.

## Data Availability

The data presented in this study are available on request from the corresponding author.

## References

[1] Brown S.M., Doom J.R., Lechuga-Peña S., Watamura S.E., Koppels T. (2020). Stress and parenting during the global COVID-19 pandemic. Child Abus. Negl..

[2] He M., Cabrera N.J., Renteria J., Chen Y., Alonso A., McDorman A.S., Kerlow M., Reich S. (2021). Family functioning in the time of COVID-19 among economically vulnerable families: Risks and protective factors. Front. Psychol..

[3] Kalil A., Mayer S., Shah R. (2020). Impact of the COVID-19 Crisis on Family Dynamics in Economically Vulnerable Households.

[4] Magson N.R., Freeman J.Y.A., Rapee R.M., Richardson C.E., Oar E.L., Fardouly J. (2021). Risk and protective factors for prospective changes in adolescent mental health during the COVID-19 pandemic. J. Youth Adolesc..

[5] Pachter L.M., Coll C.G., Perez-Brena N.J., Lopez L.M., Halgunseth L.C., Mistry R.S., Stein G.L., Carlo G. (2020). Assessing the impact of COVID-19 on children and youth. Del. J. Public Health.

[6] De Figueiredo C.S., Sandre P.C., Portugal L.C.L., Mázala-de-Oliveira T., da Silva Chagas L., Raony Í., Ferreira E.S., Giestal-de-Araujo E., dos Santos A.A., Bomfim P.O.S. (2021). COVID-19 pandemic impact on children and adolescents’ mental health: Biological, environmental, and social factors. Prog. Neuro-Psychopharmacol. Biol. Psychiatry.

[7] United States Census Bureau https://www.census.gov/library/stories/2020/09/poverty-rates-for-blacks-and-hispanics-reached-historic-lows-in-2019.html#:~:text=Poverty%20rates%20in%202019%20were,low%20of%2017.6%25%20in%202018.

[8] Pew Research Center https://www.pewresearch.org/race-ethnicity/2021/07/15/many-hispanics-have-had-covid-19-or-lost-someone-to-it/.

[9] National Research Center on Hispanic Children & Families https://www.hispanicresearchcenter.org/research-resources/latino-child-poverty-rose-during-the-covid-19-pandemic-especially-among-children-in-immigrant-families/.

[10] Pew Research Center https://www.pewsocialtrends.org/2020/04/21/about-half-of-lower-income-americans-report-household-job-or-wage-loss-due-to-covid-19/.

[11] Canady V.A. (2020). APA stress report amid COVID-19 points to parental challenges. Ment. Health Wkly..

[12] Conger R.D., Conger K.J., Martin M.J. (2010). Socioeconomic status, family processes, and individual development. J. Marriage Fam..

[13] Fontanesi L., Marchetti D., Mazza C., Di Giandomenico S., Roma P., Verrocchio M.C. (2020). The effect of the COVID-19 lockdown on parents: A call to adopt urgent measures. Psychol. Trauma Theory Res. Pract. Policy.

[14] Neppl T.K., Senia J.M., Donnellan M.B. (2016). Effects of economic hardship: Testing the family stress model over time. J. Fam. Psychol..

[15] Masten A.S. (2001). Ordinary magic: Resilience processes in development. Am. Psychol..

[16] Masten A., Barnes A. (2018). Resilience in children: Developmental perspectives. Children.

[17] Beharie N., Mercado M., McKay M. (2017). A protective association between SNAP participation and educational outcomes among children of economically strained households. J. Hunger Environ. Nutr..

[18] Cabrera N.J., Beeghly M., Brown C., Casas J., Palacios N., Phinney J., Rodriguez M., Rowley S., Santos C., SRCD Committee (2013). Positive development of minority children. Soc. Policy Rep..

[19] Masten A.S., Reed M.G.J., Snyder C.R., Lopez S.J. (2002). Resilience in development. Handbook of Positive Psychology.

[20] Li S., Xu Q. (2020). Family support as a protective factor for attitudes toward social distancing and in preserving positive mental health during the COVID-19 pandemic. J. Health Psychol..

[21] Schug C., Morawa E., Geiser F., Hiebel N., Beschoner P., Jerg-Bretzke L., Albus C., Weidner K., Steudte-Schmiedgen S., Borho A. (2021). Social support and optimism as protective factors for mental health among 7765 healthcare workers in Germany during the COVID-19 pandemic: Results of the VOICE study. Int. J. Environ. Res. Public Health.

[22] Harvey J., Delfabbro P. (2004). Psychological resilience in disadvantaged youth: A critical overview. Aust. Psychol..

[23] Chazan-Cohen R., Raikes H., Brooks-Gunn J., Ayoub C., Pan B.A., Kisker E.E., Roggman L., Fuligni A.S. (2009). Low-income children’s school readiness: Parent contributions over the first five years. Early Educ. Dev..

[24] Cheung R.Y.M., Cheng W.Y., Li J.B., Lam C.B., Chung K.K.H. (2021). Parents’ depressive symptoms and child adjustment: The mediating role of mindful parenting and children’s self-regulation. Mindfulness.

[25] Murry V.M., Bynum M.S., Brody G.H., Willert A., Stephens D. (2001). African American single mothers and children in context: A review of studies on risk and resilience. Clin. Child Fam. Psychol. Rev..

[26] Olson S.L., Ceballo R., Park C. (2002). Early problem behavior among children from low-income, mother-headed families: A multiple risk perspective. J. Clin. Child Adolesc. Psychol..

[27] Cabrera N.J., Fagan J., Wight V., Schadler C. (2011). Influence of mother, father, and child risk on parenting and children’s cognitive and social behaviors: Influence of mother, father, and child risk. Child Dev..

[28] Griffith D.M., Sharma G., Holliday C.S., Enyia O.K., Valliere M., Semlow A.R., Stewart E.C., Blumenthal R.S. (2020). Men and COVID-19: A biopsychosocial approach to understanding sex differences in mortality and recommendations for practice and policy interventions. Prev. Chronic Dis..

[29] Romero E., López-Romero L., Domínguez-Álvarez B., Villar P., Gómez-Fraguela J.A. (2020). Testing the effects of COVID-19 confinement in Spanish children: The role of parents’ distress, emotional problems and specific parenting. Int. J. Environ. Res. Public Health.

[30] Duncan G.J., Murnane R.J. (2016). Rising inequality in family incomes and children’s educational outcomes. Russell Sage Found. J. Soc. Sci..

[31] Masarik A.S., Conger R.D. (2017). Stress and child development: A review of the Family Stress Model. Curr. Opin. Psychol..

[32] Rodriguez C.M., Lee S.J., Ward K.P., Pu D.F. (2021). The perfect storm: Hidden risk of child maltreatment during the Covid-19 pandemic. Child Maltreatment.

[33] Hertz-Palmor N., Moore T.M., Gothelf D., DiDomenico G.E., Dekel I., Greenberg D.M., Brown L.A., Matalon N., Visoki E., White L.K. (2021). Association among income loss, financial strain and depressive symptoms during COVID-19: Evidence from two longitudinal studies. J. Affect. Disord..

[34] Lawson M., Piel M.H., Simon M. (2020). Child maltreatment during the COVID-19 pandemic: Consequences of parental job loss on psychological and physical abuse towards children. Child Abus. Negl..

[35] Masten A.S. (2018). Resilience theory and research on children and families: Past, present, and promise. J. Fam. Theory Rev..

[36] Masten A.S., Zelazo P.D. (2013). Risk and resilience in development. The Oxford Handbook of Developmental Psychology.

[37] Masten A.S., Cicchetti D., Cicchetti D. (2016). Resilience in development: Progress and transformation. Developmental Psychopathology.

[38] Shonkoff J.P., Phillips D.A. (2000). From Neurons to Neighborhoods: The Science of Early Childhood Development.

[39] Lewandowski A.S., Palermo T.M., Stinson J., Handley S., Chambers C.T. (2010). Systematic review of family functioning in families of children and adolescents with chronic pain. J. Pain.

[40] Caspi A., Moffitt T.E., Morgan J., Rutter M., Taylor A., Arseneault L., Tully L., Jacobs C., Kim-Cohen J., Polo-Tomas M. (2004). Maternal expressed emotion predicts children’s antisocial behavior problems: Using monozygotic-twin differences to identify environmental effects on behavioral development. Dev. Psychol..

[41] Smith K.E., Landry S.H., Swank P.R. (2000). The Influence of early patterns of positive parenting on children’s preschool outcomes. Early Educ. Dev..

[42] Roos L.E., Salisbury M., Penner-Goeke L., Cameron E.E., Protudjer J.L.P., Giuliano R., Afifi T.O., Reynolds K. (2021). Supporting families to protect child health: Parenting quality and household needs during the COVID-19 pandemic. PLoS ONE.

[43] Burke L. (2003). The impact of maternal depression on familial relationships. Int. Rev. Psychiatry.

[44] Jackson P.B., Erving C.L. (2020). Race-ethnicity, social roles, and mental health: A research update. J. Health Soc. Behav..

[45] Russell B.S., Hutchison M., Tambling R., Tomkunas A.J., Horton A.L. (2020). Initial challenges of caregiving during COVID-19: Caregiver burden, mental health, and the parent-child relationship. Child Psychiatry Hum. Dev..

[46] Calvano C., Engelke L., Di Bella J., Kindermann J., Renneberg B., Winter S.M. (2021). Families in the COVID-19 pandemic: Parental stress, parent mental health and the occurrence of adverse childhood experiences—results of a representative survey in Germany. Eur. Child Adolesc. Psychiatry.

[47] Sroufe L.A. (2005). Attachment and development: A prospective, longitudinal study from birth to adulthood. Attach. Hum. Dev..

[48] Eisenberg N., Fabes R.A., Balter L., Tamis-LeMonda C.S. (2006). Emotion regulation and children’s socioemotional competence. Child Psychology: A Handbook of Contemporary Issues.

[49] Belsky J. (1990). Parental and nonparental child care and children’s socioemotional development: A decade in review. J. Marriage Fam..

[50] Evans G.W., Li D., Whipple S.S. (2013). Cumulative risk and child development. Psychol. Bull..

[51] Fiorini M., Keane M.P. (2014). How the allocation of children’s time affects cognitive and noncognitive development. J. Labor Econ..

[52] Cappa K.A., Begle A.M., Conger J.C., Dumas J.E., Conger A.J. (2011). Bidirectional relationships between parenting stress and child coping competence: Findings from the Pace study. J. Child Fam. Stud..

[53] Crnic K., Ross E., Deater-Deckard K., Panneton R. (2017). Parenting stress and parental efficacy. Parental Stress and Early Child Development.

[54] De Cock E.S.A., Henrichs J., Klimstra T.A., Janneke B.M., Maas A., Vreeswijk C.M.J.M., Meeus W.H.J., van Bakel H.J.A. (2017). Longitudinal associations between parental bonding, parenting stress, and executive functioning in toddlerhood. J. Child Fam. Stud..

[55] Rollè L., Prino L.E., Sechi C., Vismara L., Neri E., Polizzi C., Trovato A., Volpi B., Molgora S., Fenaroli V. (2017). Parenting stress, mental health, dyadic adjustment: A structural equation model. Front. Psychol..

[56] Davis A.N., Carlo G., Crockett L.J. (2020). The role of economic stress in parents’ depression and warmth and adolescents’ prosocial behaviors among U.S. Latino/as. Peace Confl. J. Peace Psychol..

[57] Davis A.N., Carlo G., Streit C., Crockett L.J. (2018). Considering economic stress and empathic traits in predicting prosocial behaviors among U.S. Latino adolescents. Soc. Dev..

[58] Padilla-Walker L.M., Carlo G., Padilla-Walker L.M., Carlo G. (2014). The study of prosocial behavior. Prosocial Development: A Multidimensional Approach.

[59] Reich S.M., Dahlin M., Tulagan N., Kerlow M., Cabrera N., Piroutek M.J., Heyming T. (2021). Caregivers’ experiences during the COVID-19 pandemic and their children’s behavior. J. Fam. Issues.

[60] Calderón-Tena C.O., Knight G.P., Carlo G. (2011). The socialization of prosocial behavioral tendencies among Mexican American adolescents: The role of familism values. Cult. Divers. Ethn. Minority Psychol..

[61] Zucker E., Howes C. (2009). Respectful relationships: Socialization goals and practices among Mexican mothers. Infant Ment. Health J..

[62] Lee S.J., Ward K.P., Chang O.D., Downing K.M. (2021). Parenting activities and the transition to home-based education during the COVID-19 pandemic. Child. Youth Serv. Rev..

[63] Wright M.O., Masten A.S., Narayan A.J., Goldstein S., Brooks R.B. (2013). Resilience processes in development: Four waves of research on positive adaptation in the context of adversity. Handbook of Resilience in Children.

[64] Masten A.S. (2011). Resilience in children threatened by extreme adversity: Frameworks for research, practice, and translational synergy. Dev. Psychopathol..

[65] Vanderbilt-Adriance E., Shaw D.S. (2008). Protective factors and the development of resilience in the context of neighborhood disadvantage. J. Abnorm. Child Psychol..

[66] Assad K.K., Donnellan M.B., Conger R.D. (2007). Optimism: An enduring resource for romantic relationships. J. Personal. Soc. Psychol..

[67] Baumgartner J.N., Schneider T.R., Capiola A. (2018). Investigating the relationship between optimism and stress responses: A biopsychosocial perspective. Personal. Individ. Differ..

[68] Brissette I., Scheier M.F., Carver C.S. (2002). The role of optimism in social network development, coping, and psychological adjustment during a life transition. J. Personal. Soc. Psychol..

[69] Carver C.S., Johnson S.L., Joormann J. (2008). Serotonergic function, two-mode models of self-regulation, and vulnerability to depression: What depression has in common with impulsive aggression. Psychol. Bull..

[70] Kochanska G., Aksan N., Penney S.J., Boldt L.J. (2007). Parental personality as an inner resource that moderates the impact of ecological adversity on parenting. J. Personal. Soc. Psychol..

[71] Taylor Z.E., Larsen-Rife D., Conger R.D., Widaman K.F., Cutrona C.E. (2010). Life stress, maternal optimism, and adolescent competence in single mother, African American families. J. Fam. Psychol..

[72] Taylor Z.E., Widaman K.F., Robins R.W., Jochem R., Early D.R., Conger R.D. (2012). Dispositional optimism: A psychological resource for Mexican-origin mothers experiencing economic stress. J. Fam. Psychol..

[73] Jeon S., Neppl T.K. (2019). Economic pressure, parent positivity, positive parenting, and child social competence. J. Child Fam. Stud..

[74] Feinberg M.E. (2003). The Internal structure and ecological context of coparenting: A framework for research and intervention. Parenting.

[75] McHale J.P. (2007). When infants grow up in multiperson relationship systems. Infant Ment. Health J..

[76] Cabrera N.J., Scott M., Fagan J., Steward-Streng N., Chien N. (2012). Coparenting and children’s school readiness: A mediational model. Fam. Process.

[77] Choi J., Becher E.H. (2018). Supportive coparenting, parenting stress, harsh parenting, and child behavior problems in nonmarital families. Fam. Process.

[78] Mack R.A., Gee C.B. (2018). African American and Latina adolescent mothers’ and their children’s fathers’ reports of coparenting and child behavior problems: Child gender as a moderator. J. Child Fam. Stud..

[79] Morrill M.I., Hines D.A., Mahmood S., Córdova J.V. (2010). Pathways between marriage and parenting for wives and husbands: The role of coparenting. Fam. Process.

[80] Palkovitz R., Fagan J., Hull J. (2013). Coparenting and children’s well-being. Handbook of Father Involvement: Multidisciplinary Perspectives.

[81] McRae C.S., Overall N.C., Henderson A.M.E., Low R.S.T., Chang V.T. (2021). Parents’ distress and poor parenting during a COVID-19 lockdown: The buffering effects of partner support and cooperative coparenting. Dev. Psychol..

[82] Frongillo E.A., Jyoti D.F., Jones S.J. (2006). Food stamp program participation is associated with better academic learning among school children. J. Nutr..

[83] Gassman-Pines A., Bellows L. (2015). SNAP recency and educational outcomes. SSRN.

[84] Duncan G.J., Morris P.A., Rodrigues C. (2011). Does money really matter? Estimating impacts of family income on young children’s achievement with data from random-assignment experiments. Dev. Psychol..

[85] Kugbey N., Meyer-Weitz A., Oppong Asante K. (2019). Access to health information, health literacy and health-related quality of life among women living with breast cancer: Depression and anxiety as mediators. Patient Educ. Couns..

[86] Grindal T., Bowne J.B., Yoshikawa H., Schindler H.S., Duncan G.J., Magnuson K., Shonkoff J.P. (2016). The added impact of parenting education in early childhood education programs: A meta-analysis. Child. Youth Serv. Rev..

[87] Plantin L., Daneback K. (2009). Parenthood, Information and Support on the Internet. A literature review of research on parents and professionals online. BMC Fam. Pract..

[88] McKee K., Cabrera N., Alonso A., Turcios M., Reich S. (2021). Determinants of fathers’ and mothers’ involvement in a parenting intervention. Psychol. Men Masc..

[89] Briggs-Gowan M.J., Carter A.S., Irwin J.R., Wachtel K., Cicchetti D.V. (2004). The Brief Infant-Toddler Social and Emotional Assessment: Screening for social-emotional problems and delays in competence. J. Pediatr. Psychol..

[90] Cohen S., Kamarck T., Mermelstein R. (1983). A global measure of perceived stress. J. Health Soc. Behav..

[91] Brailovskaia J., Margraf J. (2020). Decrease of well-being and increase of online media use: Cohort trends in German university freshmen between 2016 and 2019. Psychiatry Res..

[92] Caprara G.V., Alessandri G., Eisenberg N., Kupfer A., Steca P., Caprara M.G., Yamaguchi S., Fukuzawa A., Abela J. (2012). The positivity scale. Psychol. Assess..

[93] Feinberg M.E., Brown L.D., Kan M.L. (2012). A multi-domain self-report measure of coparenting. Parenting.

[94] Hayes A.F. (2012). Process: A Versatile Computational Tool for Observed Variable Mediation, Moderation, and Conditional Process Modeling [White Paper]. http://www.afhayes.com/public/process2012.pdf.

[95] Low N., Mounts N.S. (2022). Economic stress, parenting, and adolescents’ adjustment during the COVID-19 pandemic. Fam. Relat..

[96] Centers for Disease Control and Prevention https://www.cdc.gov/coronavirus/2019-ncov/covid-data/investigations-discovery/hospitalization-death-by-race-ethnicity.html.

[97] Cohodes E.M., McCauley S., Gee D.G. (2021). Parental buffering of stress in the time of COVID-19: Family-level factors may moderate the association between pandemic-related stress and youth symptomatology. Res. Child Adolesc. Psychopathol..

